# Skin ultrasound in systemic sclerosis: past, present and exciting future

**DOI:** 10.1093/rap/rkae012

**Published:** 2024-01-25

**Authors:** Tânia Santiago, Devis Benfaremo, Gianluca Moroncini

**Affiliations:** Rheumatology Department, Centro Hospitalar e Universitário de Coimbra, Coimbra, Portugal; Faculty of Medicine, University of Coimbra, Coimbra, Portugal; Department of Clinical and Molecular Sciences, Marche Polytechnic University, Ancona, Italy; Department of Internal Medicine, Marche University Hospital, Ancona, Italy; Department of Clinical and Molecular Sciences, Marche Polytechnic University, Ancona, Italy; Department of Internal Medicine, Marche University Hospital, Ancona, Italy

**This editorial refers to the article ‘The value of shear wave elastography in diagnosis and assessment of systemic sclerosis, published by Cai *et al*., 2023;** https://doi.org/10.1093/rap/rkad075.

SSc is a chronic multisystem CTD associated with a significant individual and social burden, including high mortality [1]. Skin involvement is a hallmark feature of this disease and has a particular relevance for classification criteria, diagnosis, identification of disease subsets, prediction of disease progression and patient perception of disease impact [1]. Skin changes often result in pain, body image disfigurement and dissatisfaction and functional limitation, contributing greatly to the disease impact upon health-related quality of life and overall life satisfaction [2].

Skin involvement in SSc is one of the major clinical issues and the most suitable one for assessment of disease progression and response to treatment [3]. Therefore, we obviously need reliable and sensitive measures to allow earlier diagnosis and to monitor disease progression and response to treatment accurately. An ideal measurement tool should be able to identify SSc patients in the early stages of the disease to enable earlier diagnosis and potentially more efficient interventions.

Currently, the assessment of skin thickness is performed, both in clinical practice and in clinical trials, by means of a semi-quantitative score that consists of palpation of the skin at 17 sites to give the modified Rodnan skin score (mRSS) [3]. Despite its simplicity and fair validity, this method has several important limitations, including its dependence on rater experience and training, high intra- and inter-observer variability and low sensitivity to change [2].

Over the past four decades, US has attracted considerable interest as a tool to evaluate skin involvement in SSc patients and overcome the limitations of the mRSS. Both B-mode and shear-wave elastography US have several advantages: wide availability (at least B-mode); easy training; safe and non-invasive imaging procedures; immediate provision of results to clinicians; allowing the examination of multiple skin sites in a single session; and ability to store collected images [4]. Other potential advantages of US assessment include the continuous nature of the data, which might overcome the limited ability of the mRSS to identify subtle, but clinically important, changes. More importantly, B-mode US has demonstrated high intra- and inter-reliability, sensitivity to detect early disease and to change over time. Also, B-mode US might allow for earlier detection of skin involvement, as suggested by positive findings in apparently unaffected skin areas. Despite all these promising results, the real value of B-mode US and elastography as a correlate or surrogate of skin involvement in SSc has remained unclear, owing a number of methodological caveats and persistent knowledge gaps [4, 5].

Recently, a systematic literature review highlighted the achievements and limitations of the accumulated evidence published until 31 May 2021 [6, 7]. The data synthesis indicated that skin US examination did not fully satisfy any of the domains of the OMERACT filter. Underlying reasons included limited or absent data regarding feasibility and discrimination (i.e. sensitivity to change, clinical trial discrimination and thresholds of meaning) [6]. Additionally, this review confirmed a remarkable heterogeneity and scarcity of information in a variety of technical aspects that might have a decisive impact on the conclusions of the US studies. These include the probe frequency, number and precise definition of skin sites assessed, skin layers evaluated, scoring system used, and blinding during image acquisition and analysis [6, 7].

Based on the previous review results and expert opinion, an international group under the auspices of World Scleroderma Foundation published recommendations for the execution and reporting of skin US studies to promote standardization and harmonization of US technical procedures [4]. Importantly, two relevant works included in the systematic literature review shed light in the construct validity of US-dermal thickness and skin stiffness against skin histological findings [4].These studies used forearm skin biopsies and found moderate to good correlations between skin thickness measured by US and histological findings. However, no clear correlation could be established between histological skin thickness and US skin stiffness [4]. In one study, both US stiffness and local mRSS were strongly correlated with histological dermal collagen content [4].

More recently, Cai *et al.* [5] demonstrated a strong correlation between shear-wave elastography values and disease activity (European Scleroderma Study Group disease activity index) and skin collagen content (collagen volume fraction). These preliminary analyses of convergent validity with tissue histology are encouraging, but further work is desirable. One crucial aspect to optimize our knowledge of skin US is feasibility and, in particular, time consumption and the best trade-off in terms of validity and feasibility regarding the minimum number of skin sites to examine [4]. Cai *et al.* [5] were the first to address this issue, reporting that reducing the number of skin sites from 17 to 13 (i.e. excluding thighs and legs) did not affect the diagnostic accuracy of shear-wave elastography.

Another important aspect that has led to growing interest in the last few years is the use of ultra-high-frequency US probes (70 MHz) [8, 9]. Naredo *et al.* [8] reported that in oedematous puffy fingers, there was a significant increase in the thickness of the dermis and hypodermis, in comparison to healthy controls (Fig. 1). These observations suggest that attention should also be given to the hypodermis, at least in the very early phase of the disease.

**Figure 1.**
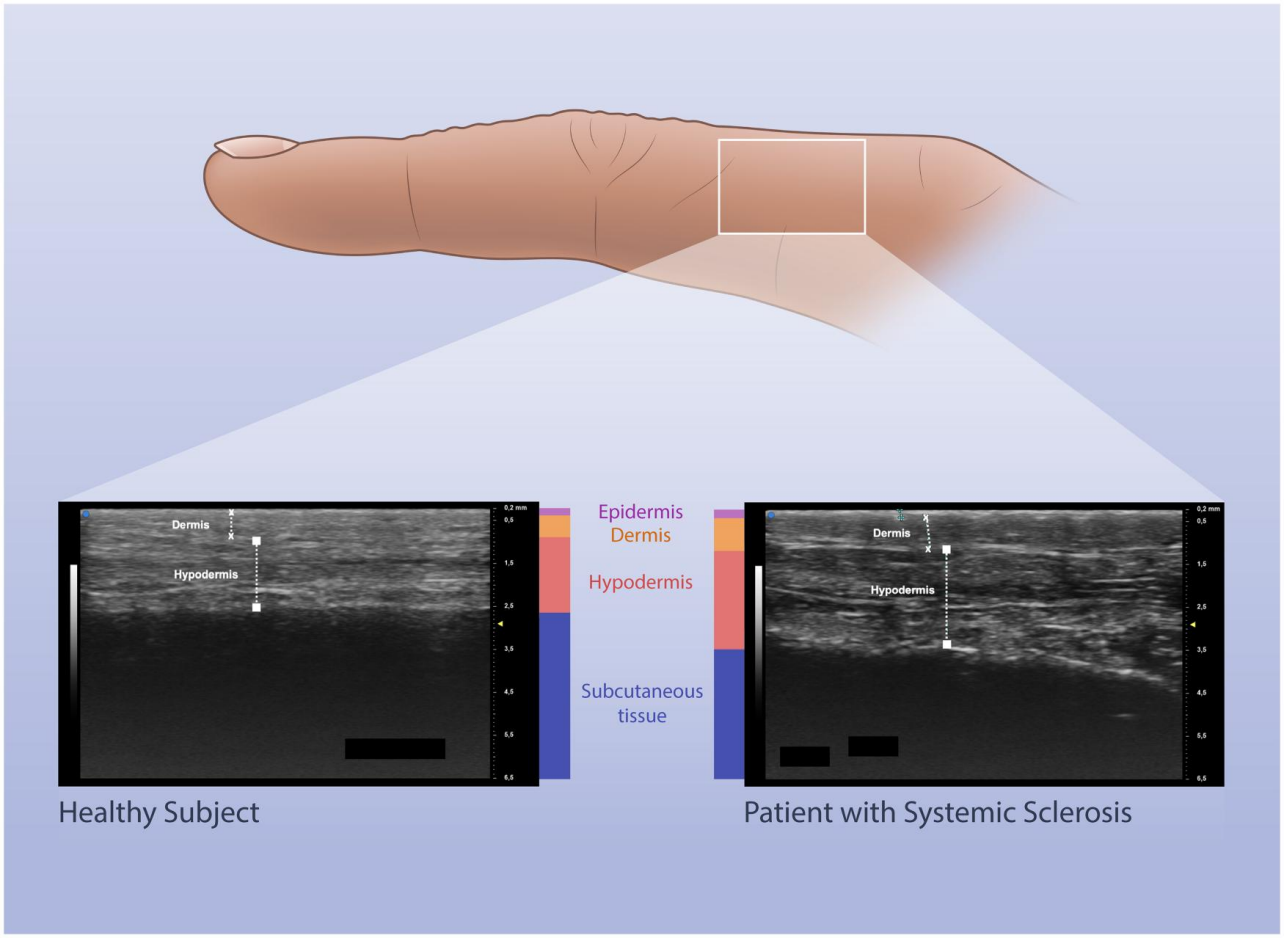
Ultrasonography of skin in SSc. Ultra-high-frequency US (70 MHz) measurement of the dermal and hypodermal thickness of the second finger of the right hand in a healthy subject (left) and in a patient with SSc (right). US images courtesy of Devis Benfaremo

There are still many unanswered questions concerning the application of US to (early) diagnosis and monitoring of skin involvement in SSc and, certainly, many new ones will emerge as new evidence becomes available. Initially, a dedicated study is needed to refine the preliminary normal reference data [10], through inclusion of heterogeneous populations from different cultural and geographical backgrounds. Then, it would be crucial to apply these normal reference values to individuals with early or undifferentiated disease at risk of SSc and ensure their follow-up as the disease progresses. In addition, it is also important to establish the sensitivity to change over time and to identify the smallest detectable change and minimal clinically important difference for US thickness and stiffness, with stratification based on disease subsets. The integration of skin US evaluation in future randomized clinical trials as a secondary or exploratory endpoint would represent a crucial step to define sensitivity to change and its clinical validity.

Finally, further collaborative studies to validate the use of US in skin and in other organ assessments (e.g. vascular, calcinosis, digital ulcers and interstitial lung disease) will definitely contribute to improve daily clinical practice and research settings in SSc.

## Data Availability

No new data were generated or analysed in support of this article.
